# The Medical Library Association Data Services Competency: a framework for data science and open science skills development

**DOI:** 10.5195/jmla.2020.909

**Published:** 2020-04-01

**Authors:** Lisa Federer, Erin Diane Foster, Ann Glusker, Margaret Henderson, Kevin Read, Shirley Zhao

**Affiliations:** National Library of Medicine, National Institutes of Health, Bethesda, MD, lisa.federer@nih.gov, https://orcid.org/0000-0001-5732-5285; Ruth Lilly Medical Library, Indiana University School of Medicine, Indianapolis, IN, erdfost@iu.edu, https://orcid.org/0000-0001-6908-9849; UC Berkeley Library, University of California Berkeley, Berkeley, CA, glusker@berkeley.edu, https://orcid.org/0000-0002-2852-5293; SDSU Library, San Diego State University, San Diego, CA, margaret.henderson@sdsu.edu, https://orcid.org/0000-0001-6578-1766; Leslie & Irene Dubé Health Sciences Library, University of Saskatchewan, Saskatoon, SK, Canada, kevin.read@usask.ca, https://orcid.org/0000-0002-7511-9036; Spencer S. Eccles Health Sciences Library, University of Utah, Salt Lake City, UT, shirley.zhao@utah.edu, https://orcid.org/0000-0003-1013-1533

## Abstract

Increasingly, users of health and biomedical libraries need assistance with challenges they face in working with their own and others’ data. Librarians have a unique opportunity to provide valuable support and assistance in data science and open science but may need to add to their expertise and skill set to have the most impact. This article describes the rationale for and development of the Medical Library Association Data Services Competency, which outlines a set of five key skills for data services and provides a course of study for gaining these skills.

Biomedical and health sciences researchers, clinicians, students, and the general public are increasingly faced with unprecedented volumes of data, necessitating a level of data literacy that enables them to effectively transform data into knowledge. A move toward more open science has also led to new ways of disseminating research outputs that reach a wider range of people. As trusted providers of information who engage with users throughout the research and information-seeking process, librarians are well-suited to support users in enhancing their data literacy skills and to help users work effectively with various types of data and communicate their science more openly. Many of the skills that librarians apply to managing scholarly literature are also relevant for data, such as curation, preservation, and access.

The Medical Library Association (MLA) has begun to explore how to help librarians gain new knowledge and skills to support users’ data needs. While some training resources will need to be developed, many courses already exist to help librarians gain data-related skills and expertise. However, with many courses being available and many skills emerging as relevant to library data services, determining how to best focus one’s continuing education can be difficult. Here, the authors introduce a “Data Services Competency” framework to guide librarians who are pursuing training to enhance their data and open science skills. This framework will also inform the development of new training opportunities to meet professional development needs.

## METHODS

A working group of librarians who had experience in providing data services was convened in October 2018 with the goal of developing an MLA competency [1] comprising the skills and knowledge that are most relevant to data librarianship today. In addition, a separate review group of librarians who were experienced in providing data support was formed to give feedback on the working group’s proposed competency.

The working group chose the name “Data Services” for the competency because it was inclusive of the variety of activities performed by health information professionals who provide data support. Next, the working group outlined the scope of the competency and revised it based on feedback from the review group. The resulting scope statement provides an overview of the range of activities that librarians may engage in to support data and open science:

We have a range of data-related knowledge and skills that support our users. We also understand the unique needs of our users and our institutions. We apply our skills to provide, facilitate access to, and evaluate data services, including data literacy instruction, data management, curation, preservation, visualization, analysis, sharing, and reuse. We encourage the use of open science practices where possible and the promotion of scientific practices that will ensure research integrity, reproducibility, and other ethical data practices.

Although this scope statement primarily highlights librarians’ role in supporting users, these activities also apply to working with their own data. Many librarians have their own research data, and libraries generate data in the course of their day-to-day workings, such as circulation data.

The working group then developed a set of performance indicators detailing the specific skills and knowledge that are needed for data services after reviewing a set of thirteen articles and reports **[**2–14**]** to develop an evidence base. Each document was reviewed by two working group members, who independently created a list of the skills described in the document. These skills were recorded in a data extraction document, resulting in a list of 464 skills. Because many of the listed skills were similar to each other, the working group chair (Federer) reviewed and grouped similar entries, arriving at 41 unique skills. The supplemental appendix contains the final list of skills, organized into 5 broad categories: data skills; programming, software, and technology skills; scientific skills; librarianship skills; and interpersonal skills.

This list of skills provided a starting point for developing performance indicators based on the profession’s current understanding of data services. The working group members individually drafted performance indicators, which were then refined to a set of five performance indicators with descriptions of proficiencies at the “Basic” and “Expert” levels. These performance indicators were sent to the review group for comment, and the working group incorporated the review group’s feedback into the final set of indicators presented here.

## PERFORMANCE INDICATORS

The Data Services Competency comprises five indicators that aim to cover the wide range of skills and knowledge that is needed for providing data services in a library setting. The descriptions of Basic- and Expert-level proficiency for each indicator provide additional context about the skills that librarians are expected to demonstrate at each level. The working group has aimed to achieve enough specificity to provide useful guidance, while also avoiding highly prescriptive language that would overly constrain the scope of the competency. These indicators provide guidance on general skills and knowledge, which librarians can pursue as most appropriate for the specific contexts in which they work. The group also sought to maximize the competency’s relevance over time by avoiding naming specific tools, technologies, or techniques that may become obsolete over time.

Some of the indicators included in the Data Services Competency are also relevant to activities that librarians conduct outside of data-related work. For example, many of the same general instructional design techniques that are needed for performance indicator 5 (“Provides training and consultation for data-related topics”) are relevant to librarians who do not work with data. Therefore, this set of indicators is not necessarily unique to conducting data services, but rather representative of the types of skills librarians would likely apply in such work. This list should also not be considered exhaustive; while it provides a foundation for developing and delivering data services, other skills and knowledge may be needed to provide the specific services that are most relevant to librarians’ institutions.

### Performance indicator 1: Applies principles of data literacy

The National Network of Libraries of Medicine (NNLM) takes its definition of data literacy from Ignazio and Qin, who define data literacy as “knowledge and skills involved in collecting, processing, managing, evaluating, and using data for scientific inquiry” [15, 16]. While this definition of data literacy is broadly applicable across different fields of study, disciplinary norms and standards drive the specific application of these skills.

At the Basic proficiency level, the librarian **finds, interprets, and manages data according to ethical principles.** While work on developing federated search capabilities across multiple subject-specific and generalist data repositories is ongoing, finding data sets at present requires knowledge of where data sets are deposited. In addition to locating data, librarians should be able to assess data sets and their associated metadata to determine whether a particular data set is appropriate for their users. Interpreting data does not involve formal data analysis, but rather the ability to facilitate translating an existing data set to a new use by understanding the user’s need. Finally, librarians should be familiar with skills for properly managing data sets, including file naming and versioning, preserving and storing data, and writing data management and sharing plans that comply with funder requirements [17, 18]. An understanding of ethical issues related to working with data, particularly human subjects data, is also essential.

At the Expert proficiency level, the librarian **critically appraises data and data collection methods.** Doing so requires an understanding of best practices and methodologies, which does not necessarily rise to the level of being a subject matter expert, but rather ensures that librarians can interpret documentation and methods to identify when a data set is of insufficient quality or is unsuited to the user’s purpose. The skills applied here are similar to those used in critical appraisal of literature, an activity that has long been within the scope of medical librarianship [17].

### Performance indicator 2: Establishes and advances data services

Library-based data services are relatively young; a 2012 survey found that data services were still uncommon in academic research libraries, though many respondents planned to develop such services within the next two years [19, 20]. More libraries have started offering data services since then, but given the nascent state of the field, librarians providing data services are likely launching these services from scratch. Accordingly, the ability to develop data services based on established need as well as to deepen and enhance those services over time are both necessary skills.

At the Basic proficiency level, the librarian **collects and uses knowledge of institutional and research context to initiate institutionally relevant data services.** User populations from different institutions and disciplines will have their own unique needs and norms related to working with data. Further, a library data service is part of the larger institutional setting in which groups like campus information technology, offices of sponsored research (or equivalent), and Clinical and Translational Science Award (CTSA) programs provide such services. Librarians who are aiming to establish a data service should use techniques like environmental scanning and strengths, weaknesses, opportunities, and threats (SWOT) analyses to determine unmet user needs and identify relevant institutional partners. Librarians should also have adequate institutional knowledge to understand how to adapt service models from exemplar libraries’ data services to their own institutions’ unique setting.

At the Expert proficiency level, the librarian **evaluates and expands upon existing data services by developing partnerships and becoming integrated into the research environment.** Ensuring the success of data services requires thoughtful evaluation and assessment to demonstrate that the services are correctly targeted at and meeting the needs of the intended user groups. These measures guide progression and potential expansion of services—or when appropriate, discontinuation of services that do not meet users’ needs. As the library’s data services mature, librarians may increasingly become integrated into the research environment at the institution, nurturing partnerships and collaborations with campus groups and users, and even being formally included in research projects as grant personnel.

### Performance indicator 3: Supports research data best practices across the data lifecycle

Library data services support users at various stages throughout the research process. While many library data services have focused primarily on data management, users need services across the full range of the data lifecycle, from planning prior to the start of a project to disposition of data after a project has ended. Delivering such services requires an understanding of the best practices that ensure data quality at each point in the process and for the range of research outputs, including data, software, and code.

At the Basic proficiency level, the librarian **provides guidance on generalizable, domain-agnostic research data best practices.** Librarians have opportunities to aid users in applying data best practices in a range of contexts including data management, curation, preservation, visualization, analysis, sharing, and reuse. Services that focus on generalizable best practices across the research lifecycle allow librarians to provide support to users across their institutions, regardless of disciplines or departments.

At the Expert proficiency level, the librarian **identifies and implements domain-specific research data best practices.** Many of the best practices that ensure high-quality research are applicable across disciplines, but some disciplines have unique practices for managing and working with data. The Data Curation Profiles project, for example, provides insight into how distinct disciplines use their own methods and is a useful primer for librarians working with a specific discipline [18]. Depending on researcher needs and institutional context, librarians will use this or similar resources to provide customized consultation and solutions for researchers.

### Performance indicator 4: Applies knowledge of research methods, research ethics and rigor, and open science practices

Librarians do not necessarily need advanced degrees in a field of science to be able to provide data services to users in that discipline, a finding that has been empirically demonstrated [6] and anecdotally observed among members of the working group and review group for this competency. However, while deep subject matter expertise is not required, an understanding of the methods and practices of science can help librarians provide more meaningful guidance to their users.

At the Basic proficiency level, the librarian **applies an understanding of the scientific method and ethical and sound research practices to data-related problems, encouraging open science practices when appropriate.** Delivering data services requires a basic knowledge of best practices for how data are collected, used, and reported. Even without understanding all the intricacies of the science behind the data, librarians can provide useful guidance on practices that conform with ethical standards and enhance the rigor and reproducibility of the research. An example of this can be seen in libraries’ increasing involvement in “Responsible Conduct of Research” training at their institutions [19]. Librarians are also well situated to encourage researchers to follow open science practices that help make the results of research more widely available. However, librarians should understand privacy concerns and other issues that may make open science practices impractical in some situations.

At the Expert proficiency level, the librarian **applies specialized knowledge of one or more scientific disciplines and research methods to advanced, domain-specific, data-related problems.** While some best practices are applicable across most types of data, many users face challenges unique to their disciplines, data types, or methods. For example, the challenges that clinical researchers face differ from those of basic science researchers because of the differences in data such as scope, collection methods, and involvement of human subjects. Librarians who are aiding users with more complex challenges may need deeper knowledge of the domain. The development of partnerships as described in performance indicator 2 also enable librarians to assist even if the users’ needs are more specialized, by knowing appropriate institutional resources or groups to whom to refer users.

### Performance indicator 5: Provides training and consultation for data-related topics

Some data services can be delivered without a significant amount of user interaction, such as ongoing preservation of existing data sets. However, in most cases, data services involve a significant amount of user training, whether one-on-one or in formal instructional sessions. In addition to data-related subject matter knowledge, librarians must have the skills in teaching and consultation that enable them to effectively transfer that knowledge to their users.

At the Basic proficiency level, the librarian **develops and delivers instruction to enhance data literacy and skills.** The training that librarians provide at this level would likely be broadly applicable and based upon general data literacy and data best practices. In some cases, template courses could be used as an aid for delivering general instruction [19, 20].

At the Expert proficiency level, a health information professional **provides customized discipline- and context-specific training on advanced data-related topics, including those that require computational approaches.** This customization may involve developing or adapting training to include specialized data types, data practices, and computational approaches that are most relevant to the target audience. Librarians at this level may also teach more advanced computational skills, such as coding in scientific programming languages. It should be noted that coding ability is not a prerequisite for attaining this level and is only recommended where demonstrated institutional need exists and librarians have a personal interest in gaining such skills.

## CONCLUSION AND NEXT STEPS

Information about how to obtain the MLA Data Services Competency will be available on MLA’s website later in 2020. The website will contain a list of existing courses that the working group has determined satisfy the requirements of the competency, including a detailed breakdown of which indicators and proficiency levels each course covers. Some of these courses are already approved for MLA continuing education (CE) credit, and MLA will continue working on approval for the remaining courses.

While some courses already exist, ample opportunity exists for development of additional courses that would address elements of this competency. The indicators and proficiencies outlined here can serve as guidance for librarians who are interested in developing future courses for inclusion in the competency, as well as for the MLA Curriculum Committees charged with leading CE course development.

The working group’s process considered the existing literature and state of practice to create a competency that will prepare librarians and information professionals to deliver services that address a range of data needs in the current research ecosystem. However, practices and technologies in these areas rapidly evolve, so this competency should be considered a living document rather than a static list of skills. While the working group has made an effort to develop indicators and proficiencies that will remain relevant for the foreseeable future, the group recommends that MLA and the profession continue to monitor the research ecosystem to ensure that this competency remains useful for librarians who are interested in gaining relevant and timely skills for years to come.

## SUPPLEMENTAL FILE

Appendix Skills extracted from the literature

## 

**Lisa Federer, AHIP**, lisa.federer@nih.gov, https://orcid.org/0000-0001-5732-5285, National Library of Medicine, National Institutes of Health, Bethesda, MD

**Erin Diane Foster**, erdfost@iu.edu, https://orcid.org/0000-0001-6908-9849, Ruth Lilly Medical Library, Indiana University School of Medicine, Indianapolis, IN

**Ann Glusker, AHIP**, glusker@berkeley.edu, https://orcid.org/0000-0002-2852-5293, UC Berkeley Library, University of California Berkeley, Berkeley, CA

**Margaret Henderson, AHIP**, margaret.henderson@sdsu.edu, https://orcid.org/0000-0001-6578-1766, SDSU Library, San Diego State University, San Diego, CA

**Kevin Read**, kevin.read@usask.ca, https://orcid.org/0000-0002-7511-9036, Leslie & Irene Dubé Health Sciences Library, University of Saskatchewan, Saskatoon, SK, Canada

**Shirley Zhao**, shirley.zhao@utah.edu, https://orcid.org/0000-0003-1013-1533, Spencer S. Eccles Health Sciences Library, University of Utah, Salt Lake City, UT
