# Supplementary material for: The Medical Library Association Data Services Competency: a framework for data science and open science skills development
Source: J Med Libr Assoc. 2020 Apr 1;108(2):304–9. doi: 10.5195/jmla.2020.909 (PMC7069817; doi:10.5195/jmla.2020.909)
Supplement: Appendix [file jmla-108-304-s001.pdf]

JMLA Journal of the Medical Library Association

SC

Supplemental content to
J Med Libr Assoc. Apr;108(2):dx.doi.org/10.5195/jmla.2020.909
www.jmla.mlanet.org
© Federer, Foster, Glusker Henderson, Read, Zhao 2020

The Medical Library Association Data Services Competency: a framework for data science and open science skills development

Lisa Federer, AHIP; Erin Diane Foster; Ann Glusker, AHIP; Margaret Henderson, AHIP; Kevin Read; Shirley Zhao

APPENDIX

Skills extracted from the literature

This list includes the forty-one skills extracted from thirteen articles and reports reviewed as part of the process of developing performance indicators.

Data skills

data curation

data discovery

data ethics and security

data literacy

data management

data management planning

data policy

data sharing

data storage and preservation

data structures and standards

data use and reuse

data visualization

metadata and documentation

repository support

scholarly communication and licensing

versioning and version control

Programming, software, and technology skills

extensible markup language (XML)

general computing

geographic information system (GIS)

scientific programming (including R, Python, Java, MATLAB, etc.)

statistical software

structured query language (SQL)

web development and web 2.0

web development and web technologies

JMLA Journal of the Medical Library Association
Supplemental content to
J Med Libr Assoc. Apr;108(2):dx.doi.org/10.5195/jmla.2020.909
www.jmla.mlanet.org
© Federer, Foster, Glusker Henderson, Read, Zhao 2020

Scientific skills
experimental design and research methods
subject matter expertise

Librarianship skills
general librarianship
reference skills

Interpersonal skills
commitment to lifelong learning
communication skills
ethics
evaluation
institutional awareness
interpersonal skills and relationship building
marketing and outreach
personal traits
problem solving
project and time management
service development
strategic planning
teaching skills
